# Adsorption and Visible Photocatalytic Synergistic Removal of a Cationic Dye with the Composite Material BiVO_4_/MgAl–LDHs

**DOI:** 10.3390/ma16216879

**Published:** 2023-10-26

**Authors:** Yuquan Wang, Yidong Xu, Xinjie Cai, Jinting Wu

**Affiliations:** 1 College of Civil Engineering and Architecture, Zhejiang University, Hangzhou 310058, China; 2 College of Civil Engineering and Architecture, NingboTech University, Ningbo 315100, China

**Keywords:** BiVO_4_, MgAl–LDHs, memory effect, adsorption-visible photocatalytic performance, organic pollutants

## Abstract

Adsorption and photocatalysis are effective in removing organic pollutants from wastewater. This study is based on the memory effects of MgAl–layered double hydroxides (MgAl–LDHs) after high-temperature calcination. By introducing bismuth vanadate (BiVO_4_) during the reformation of the layered structure via contact with water, a composite material BiVO_4_/MgAl–LDHs with enhanced adsorption and visible light catalytic performance was synthesized. The effects of the calcination temperature, ratio, initial methylene blue (MB) concentration, and catalyst dosage on the adsorption and photocatalytic performance were investigated. The BiVO_4_/MgAl–LDHs showed better photocatalytic performance than the pure BiVO_4_ and MgAl–LDHs. Under the optimal conditions, the proportion of MB adsorbed in 20 min was 66.1%, and the percentage of MB degraded during 100 min of photolysis was 92.4%. The composite photocatalyst showed good chemical stability and cyclability, and the adsorption-degradation rate was 86% after four cycles. Analyses of the adsorption and photocatalytic mechanisms for the composite material showed that synergistic adsorption and visible light photocatalysis contributed to the excellent catalytic performance of the BiVO_4_/MgAl–LDHs. A highly adsorbent photocatalytic composite material exhibiting outstanding performance was prepared via a simple, cost-effective, and environmentally friendly method, providing reference information for the removal of organic pollutants from liquids.

## 1. Introduction

With the acceleration of urbanization and industrialization, the material living standards of people have continuously improved; however, urbanization and industrialization severely challenge the environment. Among instances of these processes, the discharge of dye wastewater with high toxicity, high chroma, and complex composition seriously threatens the ecological environment and human health [1,2]. In the past ten years, researchers have attempted to use multiple techniques, including biological methods, physical/chemical adsorption, and Fenton processes [3] to treat sewage; nevertheless, these traditional treatment methods cannot be widely applied due to their disadvantages, including their high cost, complexity, and poor organic-pollutant-selectivity [4]. Due to its mild reaction conditions, high degradation efficiency, and ability to avoid secondary pollution, semiconductor photocatalytic technology has played a vital role in solar energy conversion and environmental protection measures [5]. As one of the first widely used photocatalysts, TiO_2_ has the advantages of nontoxicity, low cost, high chemical stability, strong oxidative reduction, and abundant natural reserves [6,7,8,9]. However, TiO_2_ can only respond to ultraviolet light because of its large band gap (3.2 eV), and its sunlight utilization rate is low [10]. In addition, the photocatalytic performance of TiO_2_ is reduced due to the rapid recombination of photogenerated carriers [11], limiting its real-world applicability. To overcome these shortcomings, improving the conversion of solar energy and the degradation rate of pollutants has become the focus of research in the field of photocatalysis. Notably, the proportion of ultraviolet light in sunlight is only 4–6%, while the proportion of visible light can reach 45% [8]. Therefore, the successful design of photocatalysts that can respond to visible light is becoming a popular subject among researchers.

Recent studies have confirmed that among many visible–light–metal–oxide–photocatalysts, bismuth vanadate (BiVO_4_) is a highly promising visible light catalyst [12]. The high abundance and narrow bandgap (approximately 2.4 eV) of BiVO_4_ make it vital for the photocatalytic degradation of organic matter [13,14]. However, the narrow bandgap facilitates recombination of the photogenerated electrons and holes [15]. Add–tionally, the low specific surface area of the pore–free structure and low light absorption also inhibit the photocatalytic reactions [16]. To enhance the light absorption efficiency and suppress the recombination of photogenerated electrons and holes, modifications of BiVO_4_ with other materials have attracted considerable attention [17,18,19]. Among the modification methods, the construction of heterojunctions is considered the most effective [20]. Although the heterojunctions effectively mitigate recombination of the photogenerated charge carriers, the adsorptivities of the photocatalytic composite materials are not high. Moreover, they do not exhibit efficient photocatalysis with insufficient light or in the dark, thus limiting the effective removal of organic pollutants. However, studies have shown that [21,22] composite photocatalytic materials formed by combining Bi–VO_4_ with strongly adsorptive materials can overcome the challenges of unfavorable environmental conditions and realize efficient removal of organic pollutants.

Layered double hydroxides (LDHs), also known as hydrotalcites, have the general formula [M_1−x_^2+^ M_x_^3+^ (OH)_2_] [Ax_/n_]^n−^·mH_2_O, where M^2+^, M^3+^, and A^n−^ represent divalent cations, trivalent cations, and interlayer anions [23]. The unique structures of LDHs are formed by electrostatic interactions between positively charged brucite-like octahedra and interlayer anions [24]. Due to their stable layered structures, large specific surface areas, strong adsorption capacities, and tunable band gaps, LDHs have attracted significant attention in photocatalysis [25,26,27]; they are ideal photocatalysts [28], catalyst supports [29], and adsorbents [16]. As photocatalysts, LDHs doped with transition metal elements are high-performance heterogeneous materials [28,30]. Furthermore, the LDHs can serve as both carriers and co-catalysts. When metals, metal oxides, or non-metal materials are dispersed within the LDH layered structure, a synergistic effect between the two materials can be achieved [31]. Additionally, when combined with other semiconductor materials, the MgAl–layered double hydroxide (MgAl–LDH) particles can serve as a barrier layer for electrons and capture site recombination, leading to improved spatial charge separation [32]. Yang et al. [33] found that materials like activated carbon, bentonite, and fly ash have low adsorption capacities and are difficult to recycle, whereas LDHs exhibit strong adsorption and provide photocatalytic degradation of adsorbed organic pollutants, allowing for repeated use of the adsorbents. In addition to the aforementioned characteristics, the memory effect is also a significant feature of LDHs. Research has revealed that LDHs subjected to high-temperature calcination (LDO) exhibit a memory effect. When exposed to water, they reconstruct their parent structure through layer-by-layer rebuilding [34], which generates oxygen and metal vacancy defects in the parent structure [35]. The creation of defects reduces the band gap, expands the light absorption range, and separates the photogenerated charge carriers. This study was based on the distinctive memory effect, strong adsorption performance, and exceptional catalytic activity of MgAl–LDHs. The introduction of BiVO_4_ when MgAl–LDO was treated with water triggered a laminate reconstruction process, leading to the synthesis of a composite material, BiVO_4_/MgAl–LDHs, which exhibited strong adsorption and efficient visible light catalysis.

In this paper, we used MgAl–LDHs and BiVO_4_ as raw materials to prepare a highly adsorbent photocatalytic composite material, BiVO_4_/MgAl–LDHs; we studied its adsorption capacity and photocatalytic degradation of methylene blue (MB) while considering the effects of multiple factors, including the calcination temperature, ratio, initial MB concentration, and catalyst dosage, on photocatalytic performance. Second, X-ray dif–fraction (XRD), X-ray photoelectron spectroscopy (XPS), scanning electron microscopy (SEM), transmission electron microscopy (TEM), Brunauer-Emmett-Teller (BET), and high-resolution transmission electron microscopy (HRTEM) were used to determine the morphology, crystal structure, and chemical binding state of the photocatalyst. Various techniques, including XPS valence band analysis, ultraviolet-visible (UV–vis) spectroscopy, and photoluminescence (PL) spectroscopy, were used to determine the energy band positions and optical properties of the catalyst. Finally, we analyzed the adsorption mechanism and the photocatalytic mechanism of the BiVO_4_/MgAl–LDH composite material.

## 2. Materials and Methods

### 2.1. Materials

MgAl–LDHs and isopropanol (IPA) were purchased from Guangdong Wengjiang Chemical Reagent Co., Ltd. (Shaoguan, China). BiVO_4_ was purchased from the Shanghai Dingyun Reagent Business Department. A total of 1–4 pairs of benzoquinone (BQ) were purchased from Shanghai Aladdin Biochemical Technology Co., Ltd. (Shanghai, China). Disodium ethylenediaminetetraacetic acid (EDTA–2Na) was purchased from Shanghai Zhanyun Chemical Co., Ltd. (Shanghai, China). The MB indicator was purchased from Wuxi Yatai United Chemical Co., Ltd. (Wuxi, China). The chemical reagents and materials used in this study were not further purified, and the water used in the experiments was deionized.

### 2.2. Synthesis of Photocatalyst BiVO_4_/MgAl–LDHs

**Figure 1:**
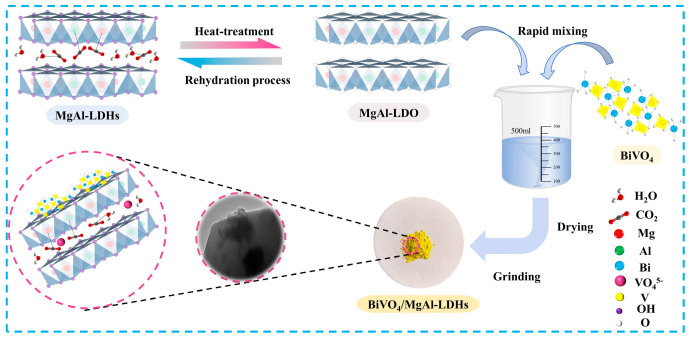
Schematic diagram showing the synthesis of BiVO_4_/MgAl–LDHs.

**Figure 2:**
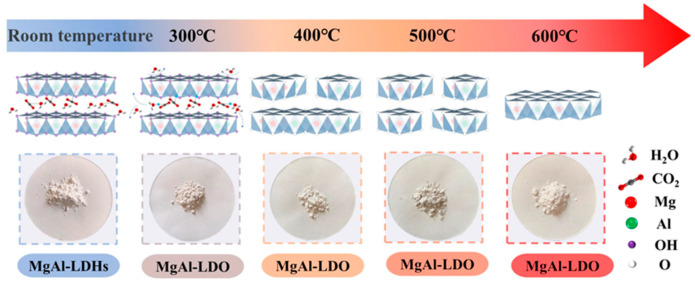
Calcination products of the MgAl–LDHs formed at different temperatures.

The synthesis of the BiVO_4_/MgAl–LDHs composite photocatalytic material mainly included the following steps (as shown in Figure 1). First, 60 g of MgAl–LDH powder was weighed and placed in a crucible. Then, the crucible was placed in the center of a muffle furnace for roasting. To explore the influences of roasting temperature, the firing temperature was set to 300 °C, 400 °C, 500 °C, and 600 °C, and the temperature was kept constant for 5 h. After the specified time, the muffle furnace was opened. After cooling to room temperature, the roasted MgAl–LDHs (MgAl–LDO) were removed. The MgAl-LDO samples prepared at various temperatures are illustrated in Figure 2. A total of 200 mL of deionized water was poured into a 1000 mL beaker. To explore the influences of different pure material ratios, BiVO_4_ and MgAl–LDO were mixed according to 2:8 (20% BiVO_4_/MgAl–LDHs), 3:7 (30% BiVO_4_/MgAl–LDHs), and 5:5 (50% BiVO_4_/MgAl–LDHs) ratios; additionally, 400 mg, 600 mg, and 1000 mg of BiVO_4_ and 1600 mg, 1400 mg, and 1000 mg of MgAl–LDO were weighed for the material ratios of 2:8, 3:7, and 5:5, respectively. Each of the above groups of BiVO_4_ and MgAl–LDO was added to a beaker containing deionized water, placed on a magnetic stirrer and stirred for 6 h. Then, the beaker was placed in a drying oven at 105 °C for drying, and the beaker was removed to cool after the drying was completed. After reaching room temperature, the sample in the beaker was ground into a powder with agate powder and sieved with a 100-mesh sieve to obtain the composite photocatalytic material BiVO_4_/MgAl–LDHs used in this study.

### 2.3. Characterization

The morphologies and structures of the photocatalysts were observed and analysed using SEM (Tescan Mira4, Energy spectrum: xplore, TESCAN Brno, s.r.o., Brno, Czech Republic) and TEM (tecnai F20, FEI Corporation, Hillsboro, OR, USA). XRD (D8 Advance, AXS Corporation, Brooke, Germany) was used to detect the material com–positions and crystal structures of the samples. UV–Vis diffuse reflection spectroscopy (Shimadzu 3600-plus, Shimadzu Corporation, Kyoto, Japan) was used to analyze the op–tical properties of the materials. Fourier transform infrared absorption spectroscopy (FTIR; Thermo Nicolet iS5, Waltham, MA, USA), XPS (American Thermo Fisher Scientific K-Alpha, Waltham, MA, USA), and steady-state/transient PL spectroscopy (Edinburgh FLS-1000, Edinburgh Instruments UK, Edinburgh, UK) were used to investigate the functional group types, surface chemical properties, and electron–hole pair recombination characteristics. The best com–posite photocatalytic material was 30% BiVO_4_/MgAl-LDH calcined at 300 °C.

### 2.4. Adsorption-Photocatalytic Degradation Experiments with MB

First, 100 mL of a 10 mg/L MB solution was prepared and poured into a beaker equipped with a stirrer. At that time, approximately 6 mL of the initial MB solution (C_0_) was extracted and filtered into a centrifuge tube. Subsequently, 500 mg of the composite material BiVO_4_/MgAl–LDHs was weighed and added to the beaker containing the MB solution. All light sources were turned off simultaneously. The beaker was then transferred to a magnetic stirrer, and the stirring speed was maintained at 270 r/min for 20 min. After stirring was completed, the photocatalytic material and MB reached adsorp–tion–desorption equilibrium. Simultaneously, about 6 mL of the MB solution (C_1_) was ex–tracted and filtered into a centrifuge tube. The light source was a 300 W xenon lamp with a 400 nm filter. The distance between the light source and the liquid surface was 45 cm, and the xenon lamp was turned on 10 min in advance for preheating. After the visible light catalytic reaction started, samples were drawn every 20 min, and the experiment ended after 100 min of light irradiation. Finally, the obtained samples were centrifuged (6000 r/min for 5 min), and the absorbance of the MB solution at different time points was measured with a 756S UV-visible spectrophotometer. Based on the relation–ship between absorbance and concentration, the concentration of the MB solution was determined at each time point.

The adsorption efficiency (R) and photocatalytic degradation rate (η_t_) for MB were calculated with Equations (1) and (2), respectively:

R = (C_0_ − C_1_)/C_0_ × 100% (1)

η_t_ = (C_1_ − C_t_)/C_1_ × 100% (2)

Adsorption–degradation rate =(C_0_ − C_t_)/C_0_ × 100% (3)

where C_0_ (mg·L^−1^) is the initial concentration of the methylene blue solution, R(%) is the MB adsorption efficiency during the dark adsorption stage, C_1_ (mg·L^−1^) is the initial con–centration of the MB solution at the start of illumination, C_t_ (mg·L^−1^) is the concentration of MB solution at time t (min) during illumination, and η_t_(%) is the photocatalytic deg–radation rate at time t.

## 3. Results and Discussion

### 3.1. Characterization of Photocatalysts

#### 3.1.1. XRD and FTIR Analysis

**Figure 3:**
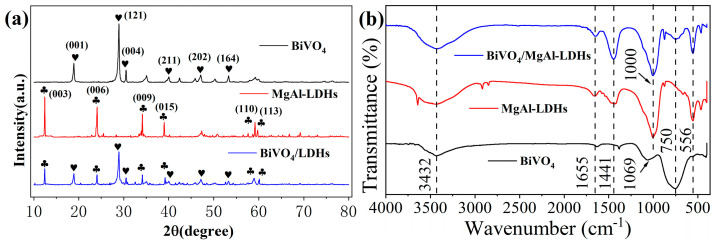
XRD patterns for BiVO_4_, MgAl–LDHs and BiVO_4_/ MgAl–LDHs (**a**) and FTIR spectra of BiVO_4_/ MgAl–LDHs, MgAl–LDHs and BiVO_4_ (**b**).

In Figure 3a, the XRD patterns of BiVO_4_, MgAl–LDHs, and BiVO_4_/MgAl–LDHs show that all of the diffraction peaks of pure BiVO_4_ are consistent with the BiVO_4_ monoclinic phase (PDF #14-0688) [36,37]. The diffraction peaks for the MgAl–LDHs exhibited high intensities and indicated distinct separation of the crystal planes, suggesting high crystallinity and regularity between the layers [38]. All of the diffraction peaks in the BiVO_4_/MgAl–LDHs XRD pattern are composed of BiVO_4_ and MgAl–LDHs; MgAl–LDHs have more diffraction peaks than BiVO_4_, which may be related to the higher proportion of MgAl–LDHs than BiVO_4_ during material synthesis. The diffraction peaks after re–combination still maintain the characteristics of the two pure materials, suggesting that the BiVO_4_/LDHs were prepared successfully.

In the FTIR spectra (Figure 3b), the peaks centered at approximately 1655 cm^−1^ and 3432 cm^−1^ of BiVO_4_ are mainly attributed to the bending and stretching vibrations of the adsorbed water molecules [39]; the peaks at 750 cm^−1^ and 1069 cm^−1^ are related to the bending vibration of VO_4_^−3^ [40] and the asymmetric tensile vibration of V–O [41], respectively. The strong and wide peak at 3432 cm^−1^ in MgAl–LDHs arises mainly due to the stretching vibrations of OH in the Mg (OH)_2_ layer, Al (OH)_3_ layer, and interlayer H_2_O. The peaks at 1655 cm^−1^, 1441 cm^−1^, and 1000 cm^−1^ originate from the bending vibrations of water molecules, the ν_3_ vibration mode of CO_3_^2^,^,^ and the translational motion of OH, respectively. The peaks near 556 cm^−1^ are interpreted as M–OH bending and M–O tensile vibration in the octahedral main layer [42]. In addition, the FTIR spectra of the composite material BiVO_4_/MgAl–LDHs exhibit the spectral characteristics of both BiVO_4_ and MgAl–LDHs, further indicating the successful preparation of the composite BiVO_4_/MgAl–LDHs.

#### 3.1.2. SEM, TEM and Energy-Dispersive X-ray Spectroscopy (EDS) Analyses

**Figure 4:**
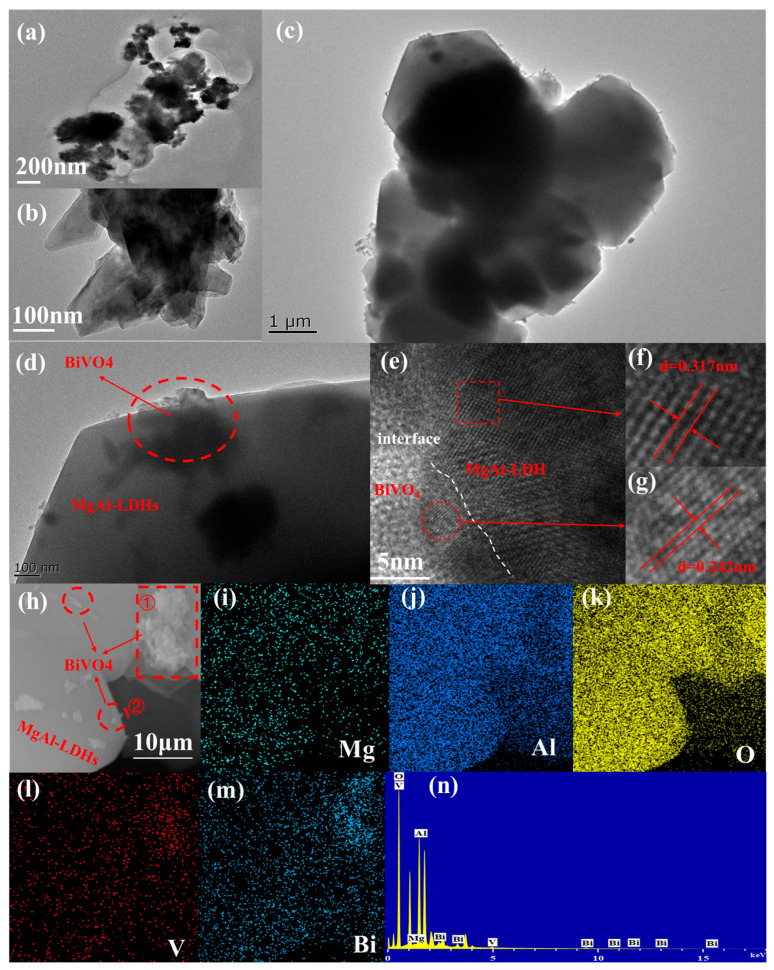
TEM images of BiVO_4_ (**a**), MgAl–LDHs (**b**), and BiVO_4_/MgAl–LDHs (**c**,**d**); HRTEM images of BiVO_4_/MgAl–LDHs (**e**–**g**); SEM–EDS elemental mapping images of BiVO_4_/MgAl–LDHs (**h**–**m**); and EDS spectrum of BiVO_4_/MgAl–LDHs (**n**).

The morphologies and microstructures of BiVO_4_, MgAl–LDHs, and BiVO_4_/MgAl–LDHs were studied by TEM. As shown in Figure 4, BiVO_4_ has an irregular block structure formed by the aggregation of particles [43] (Figure 4a) and MgAl–LDHs are hexagonal plates with certain thicknesses and surface sizes of approximately 2 µm [44] (Figure 4b). The composite material BiVO_4_/MgAl–LDHs retains the main structure of pure BiVO_4_ and MgAl–LDHs and tightly attaches BiVO_4_ to the surfaces of the hexagonal plates of MgAl–LDHs (Figure 4c,d). As shown in Figure 4e–g, the crystal plane distributions in the HRTEM images confirm the coexistence of BiVO_4_ and MgAl–LDHs, where the crys–tal plane fringe spacings of 0.317 nm and 0.242 nm correspond to the crystal plane of MgAl–LDHs and the crystal plane of BiVO_4_, respectively. The tight connection formed between these two semiconductor materials facilitates charge transfer [45]. Figure 4h shows that the distribution of BiVO_4_ in area ➁ is more uniform than that in area ➀, in–dicating that LDHs can somewhat reduce the occurrence of BiVO_4_ agglomeration and deactivation, thereby exposing an increased number of active centers. Elemental map–ping of BiVO_4_/MgAl–LDHs shows that vanadium, oxygen, bismuth, magnesium, and aluminum are evenly distributed on the surface of the photocatalyst, indicating that the heterojunction interface formed between BiVO_4_ and MgAl–LDHs is close (Figure 4i–m); Wang et al. derived similar conclusions [46]. Figure 4n shows the relationship between the elemental content of each component in BiVO_4_/MgAl–LDHs, in which the contents of oxygen and aluminum are relatively high, and the contents of bismuth and magnesi–um are relatively low.

#### 3.1.3. BET Analyses

**Figure 5:**
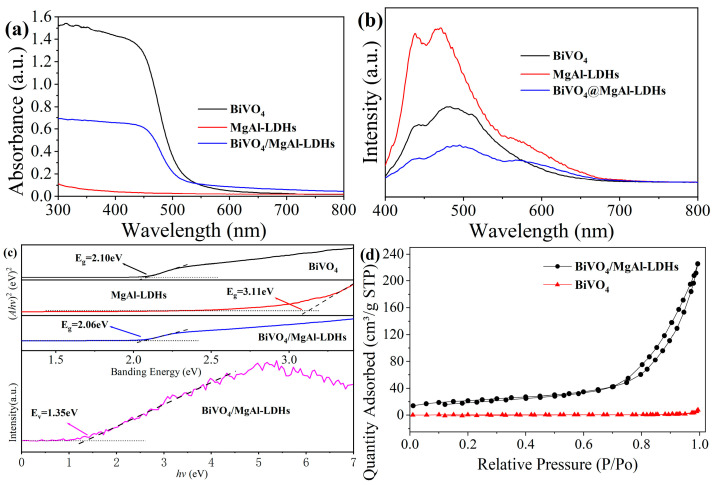
UV–vis diffuse reflectance spectra of BiVO_4_, MgAl–LDHs and BiVO_4_/MgAl–LDHs (**a**); photoluminescence spectra of BiVO_4_, MgAl–LDHs and BiVO_4_/MgAl–LDHs excited at 260 nm (**b**); optical band gaps of BiVO_4_, MgAl–LDHs and BiVO_4_ (**c**); Nitrogen sorption isotherms for BiVO_4_ and the BiVO_4_/MgAl–LDHs (**d**).

The BET surface areas (A_BET_) and pore volumes of BiVO_4_ and BiVO_4_/MgAl–LDHs were evaluated with N_2_ adsorption–desorption isotherms [47], as depicted in Figure 5d. The isotherm for BiVO_4_/MgAl–LDHs is classified as a Type V isotherm, and it exhibits a vague hysteresis loop when the P/P_0_ range is between 0.7 and 1.0. On the other hand, the isotherm for BiVO_4_ is identified as a Type II isotherm, indicating that BiVO_4_ is a non-porous material. As shown in Table 1, compared to pure BiVO_4_, the composite material BiVO_4_/MgAl–LDHs displays significantly higher A_BET_, V_BJH_, and D_BJH_ values, indicating a noticeable structural alteration upon the incorporation of the MgAl–LDH. This alteration primarily stems from the inhibited aggregation of the BiVO_4_ particles after they were loaded onto the high specific-surface-area and porous MgAl–LDHs, as highlighted in Figure 4h. 

#### 3.1.4. PL, UV–Vis–NIR Analysis

Figure 5a shows the ultraviolet–visible absorption spectra of the three samples. The composite material BiVO_4_/MgAl–LDHs has a higher absorbance than BiVO_4_ in the visible region < 542 nm and the absorbance of BiVO_4_/MgAl–LDHs is lower than that of BiVO_4_ when the wavelength is >542 nm. However, the absorbance of MgAl–LDHs is very low throughout the visible light region, further indicating that it does not respond to visible light. By taking hv as the abscissa and (Ahv)^2^ as the ordinate for plotting (Figure 5c), we find that the band gap widths of BiVO_4_/MgAl–LDHs and BiVO_4_ can be obtained as 2.09 eV and 2.05 eV, respectively. Therefore, compounding MgAl–LDHs can tune the forbidden band width of pure BiVO_4_, thereby expanding its visible light response range.

PL spectroscopy can be used to characterize the separation and transfer characteristics of photogenerated electron–hole pairs; the higher the luminescence intensity is, the higher the degree of recombination of photogenerated carriers. As shown in Figure 5b, all three materials have intensity peaks near 473 nm, among which MgAl–LDHs have the highest intensity peak, and BiVO_4_/MgAl–LDHs have the lowest intensity peak. The results show that the composite photocatalytic material BiVO_4_/MgAl–LDHs has a higher separation efficiency of photogenerated carriers than the pure materials BiVO_4_ and MgAl–LDHs. This phenomenon may arise because the positive surface charges of MgAl–LDHs capture the photogenerated electrons in BiVO_4_, thereby prolonging the lifetime of photogenerated carriers. In addition, the difference in the Fermi energy level between BiVO_4_ and MgAl–LDHs may reduce the recombination rates of photogenerated electrons and holes [48].

#### 3.1.5. XPS Analysis

**Figure 6:**
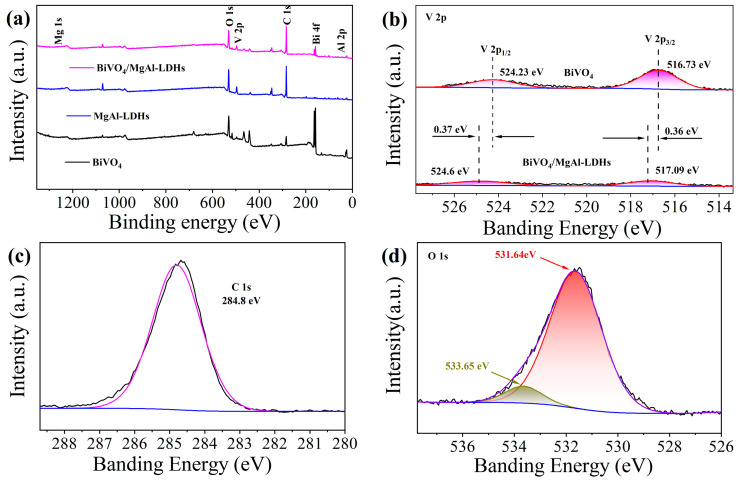

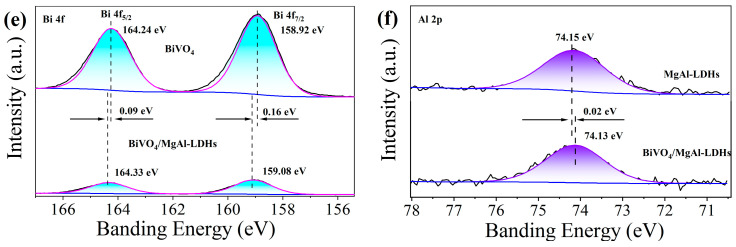
XPS survey spectra of BiVO_4_/LDHs, MgAl–LDHs and BiVO_4_ (**a**); XPS high–resolution spectra of V 2p (**b**), C 1s (**c**), O 1s (**d**), Bi 4f (**e**), and Al 2p (**f**).

XPS of BiVO_4_, MgAl–LDHs, and BiVO_4_/MgAl–LDHs was used to explore the changes in the elemental compositions and states of the sample surfaces after recombination. As shown in Figure 6a, the composite material BiVO_4_/MgAl–LDHs are mainly composed of five elements, Mg, Al, Bi, V, and O, and the contents of Mg and Bi are relatively small; this trend is consistent with the results of Figure 4n, further determining the elemental compositions of the composite BiVO_4_/MgAl–LDHs. By taking the peak at C 1s 284.8 eV as the reference standard (Figure 6c) [49], we find two sets of characteristic peaks at 531.64 eV and 533.65 eV (Figure 6d); the peak at 531.64 eV is mainly attributed to the metal oxide oxygen in the group [50], and the peak at 533.65 eV may be related to C–O–C and O–C=O in MgAl–LDHs [51]. In the XPS images of pure BiVO_4_ V 2p (Figure 6b), the two peaks at 524.23 eV and 516.73 eV belong to V 2p_1/2_ and V 2p_3/2_, respectively [52]. Compared with pure BiVO_4_, in the composite material BiVO_4_/MgAl–LDHs, the peak intensities of both V 2p_1/2_ and V 2p_3/2_ decrease, mainly because the greatly reduced low-valent V participates in the redox reaction and is consumed during composite synthesis. Second, the peaks at V 2p_1/2_ and V 2p_3/2_ move forward to 524.6 eV and 517.09 eV, respectively. The two peaks at 158.92 eV and 164.24 eV in Figure 6e represent Bi 4f_5/2_ and Bi 4f_7/2_, respectively, indicating that the ion form of Bi in the composite photocatalyst is Bi^3+^ [53]. Compared with pure BiVO_4_, Bi 4f_5/2_ and Bi 4f_7/2_ in BiVO_4_/MgAl–LDHs move in the direction of high binding energy. Here, the electron binding energies are 159.08 eV and 164.33 eV for Bi 4f_5/2_ and Bi 4f_7/2_, respectively. The above positive movement phenomenon may occur due to the deposition of BiVO_4_ on MgAl–LDHs. This deposition changes the local chemical environment so that BiVO_4_ can act as an acceptor to receive photogenerated electrons transferred from MgAl–LDHs; related studies have resulted in similar conclusions [54,55]. Contrary to the direction of movement of the abovementioned orbital electron binding energy, unlike pure MgAl–LDHs, the electron binding energy at Al 2p in BiVO_4_/MgAl–LDHs moves inversely from 74.15 to 74.13 eV (Figure 6f), which may be related to the newly formed unstable bond in the composite [56].

### 3.2. Photocatalytic Degradation of the MB Solution under Visible Light

#### 3.2.1. Effect of Firing Temperature

**Figure 7:**
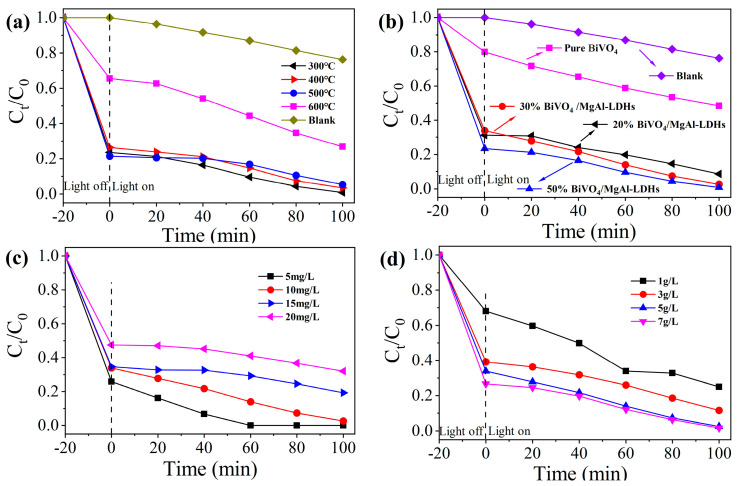
Relationship between the visible light catalytic degradation of MB and irradiation time under different calcination temperatures (**a**), ratios (**b**), initial dye concentrations (**c**), and photocatalyst concentrations (**d**).

To explore the effect of the calcination temperature of MgAl–LDHs on the adsorption of visible light and catalytic removal of MB, the products of the MgAl–LDHs were calcined at 300 °C, 400 °C, 500 °C, and 600 °C (MgAl–LDO), and a composite with BiVO_4_ was synthesized. The synthetic steps of the composite visible light catalytic materials are shown in Figure 1. The photocatalytic capabilities of these four composite materials were evaluated under identical conditions, and the results are illustrated in Figure 7a. At calcination temperatures between 300 °C and 500 °C, the adsorption efficiencies of the composite materials remain around 75%. However, as the calcination temperature increases to 600 °C, the adsorption efficiencies of the composite materials decrease to 34.6%. These substantial drops in the adsorption efficiencies are primarily attributed to the collapse and disruption of the core layered structures of the calcination products, resulting in the formation of a spinel structure with a smaller specific surface area and pore volume. Secondly, the degradation rates of the blank control group and the 300 °C, 400 °C, 500 °C, and 600 °C calcination groups are 23%, 92.4%, 86.8%, 75.0%, and 59.0%, respectively. The four composite materials show better photocatalytic performance than the control group, among which the photocatalytic effects at a calcination temperature of 300 °C are the best and at a calcination temperature of 600 °C are the poorest. This phenomenon occurs mainly because as the calcination temperature increases, the morphologies and crystal structures of the calcination products change to varying degrees. When the calcination temperature increases to 300 °C, MgAl–LDHs release H_2_O and CO_2_ successively. Based on the memory effect [57], MgAl–LDHs after calcination can still recover their layered structures in the presence of deionized water. We prepared BiVO4/MgAl–LDHs by combining MgAl–LDHs in layered reconstruction with BiVO4. When the calcination temperature increases from 300 °C to 400 °C and then to 500 °C, the MgAl–LDHs after calcination cannot recover their regular and orderly original layered structures, and their memory effects begin to weaken. Here, the degradation rate of the composite photocatalytic material begins to decrease. When the calcination temperature further increases to 600 °C, the main layered structures of the MgAl–LDHs are completely collapsed and destroyed, forming spinel structures with small specific surface areas and pore volumes; additionally, the memory effect is lost. Moreover, the degradation rate of the composite-formed photocatalytic material is the lowest. Second, when the calcination temperature is 250 °C [58] or lower [40], MgAl–LDHs lose interlayer water without significant structural changes, and their memory effects cannot be used to compound Bi–VO_4_/MgAl–LDHs; therefore, the calcination temperature of MgAl–LDHs in this study is selected as 300 °C.

#### 3.2.2. Effect of the Material Ratio

Pure BiVO_4_ easily causes accumulation and agglomeration inactivation, and the cost is overly high. An appropriate dosage of MgAl–LDHs can effectively improve the separation efficiencies of photogenerated carriers in BiVO_4_ and reduce the production costs of composite materials. BiVO_4_ and MgAl–LDHs were used to synthesize the composite materials 20% BiVO_4_/MgAl–LDHs, 30% BiVO_4_/MgAl–LDHs, and 50% BiVO_4_/MgAl–LDHs with ratios of 2:8, 3:7, and 1:1, respectively. Since MgAl–LDHs (ecbm = −0.61 eV, evbm = 2.59 eV, Eg = 3.2 eV) do not respond to visible light, we must use a blank control group, pure BiVO_4_, 20% BiVO_4_/MgAl–LDHs, 30% BiVO_4_/MgAl–LDHs, and 50% Bi–VO_4_/MgAl–LDHs to investigate the effects of the material ratio on the adsorption and photocatalytic degradation of MB (Figure 7b). When the proportion of BiVO_4_ is 20%, 30%, and 50%, the MB adsorption efficiencies during 20 min in the dark are approximately 70%. Compared to pure BiVO_4_, there is a 50% increase in the adsorption efficiency. This is attributed to (i) improved dispersion of the BiVO_4_, which exposed more active surfaces; (ii) the high specific surface area of the MgAl–LDHs provided more adsorption sites; and (iii) the interaction between BiVO_4_ and MgAl–LDHs enhanced the microstructure and surface characteristics of the composite material, as shown in Figure 5b. The degradation rates of the blank control group, pure BiVO_4_, 20% BiVO_4_/MgAl–LDHs, 30% BiVO_4_/MgAl–LDHs, and 50% BiVO_4_/MgAl–LDHs test groups are 23.4%, 39.4%, 72.3%, 92.4%, and 96.6%, respectively. The results show that the incorporation of MgAl–LDHs can improve the photocatalytic performance of BiVO_4_. As the proportion of BiVO_4_ increases (20–50%), the photocatalytic performance of the composite material gradually improves; however, the increase in the degradation rate gradually decreases. The degradation rates of 30% BiVO_4_/MgAl–LDHs and 50% BiVO_4_/MgAl–LDHs are sufficiently high to nearly completely degrade the MB solution. The above results show that incor–porating an appropriate amount of MgAl–LDHs can effectively alleviate the agglomera–tion and deactivation of BiVO_4_ and provide more active sites for the photocatalytic reaction. Second, considering the production cost of BiVO_4_ and the photocatalytic effect of the composite material, we select BiVO_4_ as the 30% composite photocatalytic material.

#### 3.2.3. Effect of Initial MB Concentration

Different MB initial concentrations (5 mg/L, 10 mg/L, 15 mg/L, and 20 mg/L) and 500 mg of 30% BiVO_4_/MgAl–LDHs were used to study the effects of the initial MB con–centration on the adsorption capacity and photocatalytic activity. The results are shown in Figure 7c. As the initial concentration of the MB solution increases from 5 mg/L to 20 mg/L, the adsorption efficiencies are 74.2%, 66.1%, 65.3%, and 52.5%, respectively. With increases in the initial MB concentration, the adsorption efficiency consistently decreases. This is primarily due to the fact that at higher initial MB concentrations, there are insufficient active adsorption sites on the surface of the composite material to adsorb all of the MB in the solution. The higher concentration of MB results in more unabsorbed MB, leading to a reduction in adsorption efficiency as the initial MB concentration was increased. The MB solution with a concentration of 5 mg/L is completely degraded when illuminated for 60 min, and the degradation rates corresponding to 10 mg/L, 15 mg/L, and 20 mg/L are 92.4%, 42.6%, and 32.5% when illuminated for 100 min, respectively. As the initial concentration of methylene blue increases, the photocatalytic degradation rate decreases continuously. This trend occurs mainly because low-concentration MB solutions can be completely degraded by sufficient active free radicals, while high-concentration MB solutions cannot be effectively decomposed and occupy a relatively high number of catalyst surface active sites, thus reducing the number of highly oxidative hydroxyl radicals [59]. Moreover, high-concentration MB solutions shorten the path lengths of photons penetrating the solution and prevent photons from reaching the surface of the photocatalyst [60]. In addition, an increasing number of sulfides, chlorides, and other intermediates adsorbed on the active site of the photocatalyst surface nega–tively impact photocatalytic degradation. To compare the photocatalytic degradation ef–fect over a long period with other experimental groups and ensure that the initial con–centration of the MB solution is not overly high, the initial concentration of MB solution selected in this study is 10 mg/L.

#### 3.2.4. Effect of Photocatalyst Dosage

A 10 mg/L methylene blue solution was used with various 30% BiVO_4_/MgAl–LDHs dosages (1 g/L, 3 g/L, 5 g/L, 7 g/L) to investigate the influence of the photocatalyst dos–age on both the adsorption capacity and photocatalytic activity. The results are shown in Figure 7d. When the catalyst dosage increases from 1 g/L to 7 g/L, the corresponding ad–sorption efficiencies are 32%, 60.8%, 66.1%, and 73.3%, respectively. The increased ad–sorption efficiencies are attributed to the greater availability of active adsorption sites [61]. Secondly, when the amount of catalyst increases from 1 g/L to 3 g/L, 5 g/L, and 7 g/L, the corresponding degradation rates are 63.3%, 70.4%, 92.4%, and 93.5%, respectively; additionally, the photocatalytic degradation rate continues to increase. The degrada–tion rate does not increase significantly when the catalyst dosage increases from 5 g/L to 7 g/L. This phenomenon occurs mainly because with the increase in the amount of pho–tocatalyst, more photogenerated electron–hole pairs and free radicals are generated and more active sites are exposed, thus improving the photocatalytic degradation efficiency [62]. However, when the amount of catalyst increases to a certain amount, the suspend–ed catalyst particles block a part of the incident light, thereby reducing the photon effi–ciency. Here, the degradation rate no longer increases or shows a downward trend [60,63]. A reasonable amount of photocatalyst is crucial for photocatalytic degradation experiments. When the amount of photocatalyst exceeds 5 g/L, the amount of photocata–lyst is no longer the main factor affecting its degradation rate; therefore, the optimal amount of photocatalyst selected in this study is 5 g/L.

#### 3.2.5. Reaction Kinetics Analysis

To investigate the effects of the calcination temperature, material ratio, initial MB concentration, and photocatalyst dosage on the visible light photocatalyst, we employed the pseudo-first-order kinetic equation [64] (as depicted in Equation (4)) to fit the exper–imental data for the visible light photocatalytic phase.

In(C_t_/C_0_) = −kt (4)

In the equation, C_0_ and C_t_ denote the initial concentration of MB at time zero and the concentration of MB at time t, both measured in mg/L. The parameter k represents the pseudo-first-order rate constant, with units of min^−1^, and time t is measured in minutes (min).

**Figure 8:**
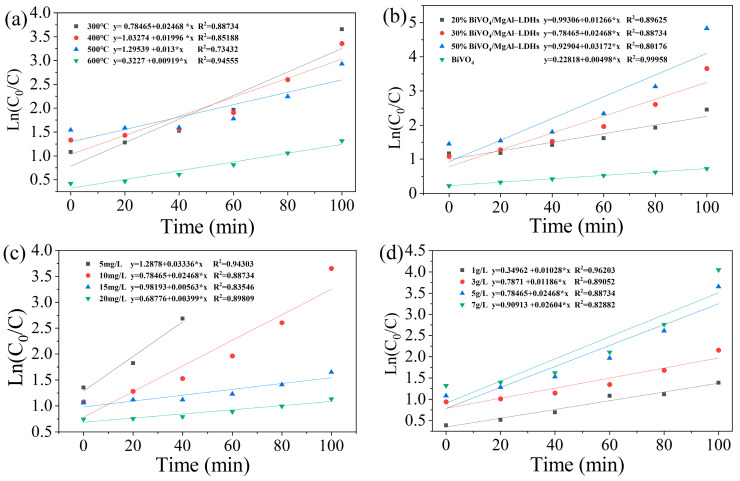
Kinetic fits for visible light catalytic degradation of MB in solution with different calcination temperatures (**a**), ratios (**b**), initial dye concentrations (**c**), and photo–catalyst concentrations (**d**).

As shown in Figure 8, the determination coefficient (R^2^) for the fit to the data con–sistently exceeded 70%, indicating the suitability of the pseudo-first-order model in rep–licating the experimental findings. Subsequently, the magnitude of the impact on visible light photocatalysis can be elucidated by comparing the fitted rate constants (k) under different conditions [46]. The rate constants associated with the varying calcination tem–peratures (Figure 8a), material ratios (Figure 8b), initial MB concentrations (Figure 8c), and photocatalyst quantities (Figure 8d) range from 0.009 to 0.025, 0.005 to 0.031, 0.004 to 0.033, and 0.010 to 0.026, respectively. Consequently, it is inferred that, in comparison to the calcination temperature and photocatalyst dose, the material ratios and initial MB concentrations have a more significant influence on the visible light photocatalytic performance.

#### 3.2.6. Reusability of Photocatalysts

**Figure 9:**
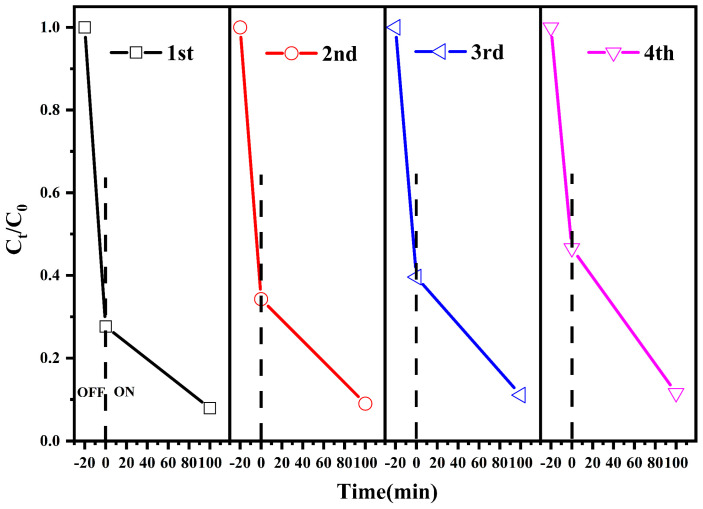
Cycling runs of the photocatalytic degradation of MB over the BiVO_4_/MgAl–LDHs pho–tocatalyst under visible light illumination.

To evaluate the chemical stability and reusability of the composite photocatalytic material BiVO_4_/MgAl–LDHs, after each experiment, the residue suspended in the beaker was allowed to stand for one day. Subsequently, the supernatant in the beaker was ex–tracted, and the photocatalytic material was washed three times with absolute ethanol. Finally, the washed photocatalytic material was transferred to a dry box for drying. This process was repeated four times with the same test conditions mentioned above. As il–lustrated in Figure 9, after four repeated tests, the adsorption–degradation rate of MB solution can reach 88.4% (only 8.7% loss), and the results show that the newly prepared photocatalytic composite has good durability and stability. In addition, studies have shown that the repeated use of photocatalysts has little effect on their physical and chemical properties [60,65]. 

### 3.3. Role of Active Species

**Figure 10:**
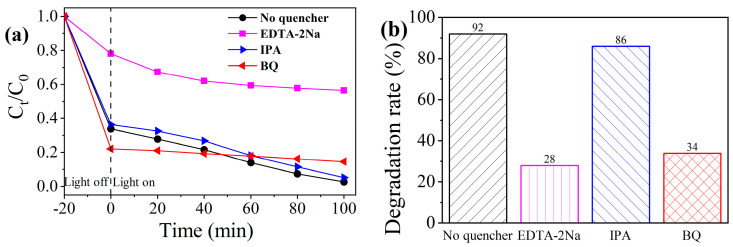
Relationship between the photocatalytic degradation of MB and irradiation time (**a**), as well as the MB degradation rates (**b**) with free radical scavengers.

To clarify the main active species in the photocatalytic reaction process, EDTA–2Na [66] was selected as the quencher of photogenerated holes (h^+^), IPA [67] was selected as the quencher of hydroxyl radicals (·OH), and BQ [68] was used as the quencher of su–peroxide radicals (·O^2−^). The photocatalytic experiment was conducted under the same experimental conditions. The results are shown in Figure 10a,b. The degradation rates corresponding to EDTA-2Na, IPA, and BQ are 28%, 86%, and 34%, respectively, and the degradation rates are 64%, 6%, and 58% lower than those of the group without a quenching agent. The results show that ·O_2_^−^ and h^+^ are the main active substances in the photocatalytic reaction process, while the effect of ·OH is relatively weak; Tang et al. drew the same conclusion [12].

### 3.4. Analysis of the Adsorption Mechanism

**Figure 11:**
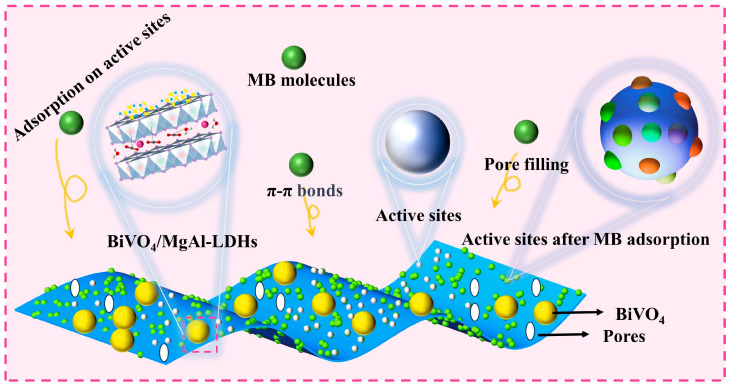
Adsorption of MB by BiVO_4_/MgAl-LDHs under dark conditions.

The study indicates that organic compounds exhibit a strong affinity for the surfaces of clay minerals [33], and as the surface area of the adsorbent increases, its adsorption capacity also increase continuously [69]. Furthermore, when MB diffuses within the composite materials with higher porosities, they are readily adsorbed onto the pore walls. From Table 1, it is evident that the composite material BiVO_4_/MgAl–LDHs has a significantly larger specific surface area and greater porosity, providing a foundation for rapid adsorption of a substantial quantity of MB in a short time. Moreover, the surface functional groups also play a crucial role in the adsorption process [61]. The adsorption of MB on BiVO_4_/MgAl–LDHs in the dark is illustrated in Figure 11. The adsorption mechanism is primarily governed by the following three modes of adsorption: (i) the agglomeration of BiVO_4_ in the composite material is ameliorated, exposing more surface active sites compared to the pure BiVO_4_. On the other hand, the MgAl–LDHs within the composite material inherently possess large specific surface areas and abundant surface active sites. Consequently, the composite material adsorbs many MB molecules at the surface active sites. (ii) During the process of pore filling in the composite material Bi–VO_4_/MgAl–LDHs, some of the MB is consumed. (iii) The free MB interacts strongly with the oxygen functional groups present in the composite material, such as OH, C–O–C, O–C=O, and forms strong π-π bonds [70] (as shown in Figure 6).

### 3.5. Analysis of the Photocatalytic Mechanism

**Figure 12:**
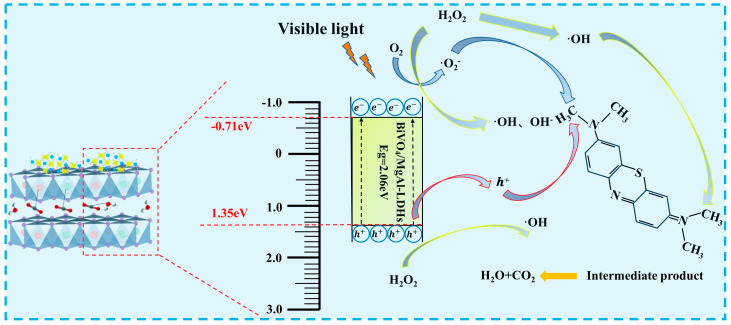
Schematic diagram showing the visible light catalytic degradation of MB by Bi–VO_4_/MgAl–LDHs.

Zeng et al. [71] discovered that photocatalytic activity is primarily related to the ad–sorption capacity of the target pollutant and the degree of separation for photogenerated electron–hole pairs. Based on the aforementioned results, it is evident that the composite material BiVO_4_/MgAl–LDHs, which was obtained by loading BiVO_4_ during the MgAl–LDH reconstruction process, exhibits strong adsorption. The oxygen and metal vacan–cies generated during the layer-by-layer reconstruction process reduces the band gap, broadens the light absorption range, and also promotes the separation of the photogen–erated charge carriers. Additionally, research has confirmed that MgAl–LDH particles can serve as a barrier layer for electrons, leading to improved spatial charge separation [32]. As depicted in Figure 5c, compared to pure BiVO_4_ and pure MgAl–LDHs, the com–posite material BiVO_4_/MgAl–LDHs exhibits a smaller band gap, indicating a broader range of visible light absorption. Figure 5c shows that the band gap and valence band potential of BiVO_4_/MgAl–LDHs are 2.06 eV and 1.35 eV, respectively. Additionally, based on the relationship between the band gap, the valence band, and the conduction band potential (Ec = E_v_ − E), the conduction band potential of BiVO_4_/MgAl–LDHs is −0.71eV. A schematic representation of the band gap energy for BiVO_4_/MgAl-LDHs is illustrated in Figure 12. The visible light degradation mechanism is shown in Equations (5)–(11) [60]. When the composite material BiVO_4_/MgAl–LDHs is excited by visible light, photogener–ated electrons in the valence band are promoted to the conduction band, leaving an equal number of photogenerated holes in the valence band. At this point, the MgAl–LDHs act as electron transfer agents, facilitating the transfer of electrons from the con–duction band to the surface of MgAl-LDHs (Equation (4)), thus reducing the rate of pho–togenerated charge carrier recombination. In the redox reactions of the photogenerated electrons and holes, O_2_ is reduced to ·O_2_^−^ and H_2_O is oxidized to ·OH. Finally, through the combined effects of various reactive species, the MB is ultimately degraded into H_2_O, CO_2_, and various intermediate products (Equations (8)–(11)), as illustrated in Figure 12 [24].

BiVO_4_/MgAl − LDHs + hv → BiVO_4_(h^+^) + MgAl − LDHs(e^−^) (5)

H_2_O + h^+^ → ·OH (6)

O_2_ + e^−^ → ·O_2_^−^ (7)

·O_2_^−^ + H_2_O → HO_2_· + OH^−^ (8)

e^−^ + HO· →H^+^ + H_2_O_2_ (9)

e^−^ + H_2_O_2_→OH· + OH^−^ (10)

·OH + MB → CO_2_+ H_2_O+ SO_4_^−^ + NO_3_^−^ + Cl^−^ (11)

## 4. Conclusions

In this study, a high-performance, highly adsorbent photocatalytic composite material, BiVO_4_/MgAl–LDHs, was prepared via a simple, cost–effective, and environmentally friendly method. In addition, considering the effects of calcination temperature, propor–tion, initial MB concentration, and photocatalyst dosage on the adsorption and photo–catalytic performance of the composite material, the following optimal photocatalytic conditions were obtained through continuous optimization: a calcination temperature of 300 °C, a ratio of 3:7 (the performance of the 50% BiVO_4_/MgAl–LDHs catalyst was slightly better than that of 30% BiVO_4_/MgAl–LDHs; considering the cost, we chose 30% Bi–VO_4_/MgAl–LDHs as the preferred photocatalyst), an initial MB concentration of 10 mg/L, and a photocatalyst dosage of 5 g/L. In addition, BiVO_4_/MgAl–LDHs showed better adsorption and photocatalytic performance than pure BiVO_4_ and MgAl–LDHs. Under the optimal conditions, the MB adsorption rate was 66.1% in 20 min, and the amount of MB degraded during 100 min of light exposure was 92.4%. Second, the composite photocatalyst had good chemical stability and repeatable practicability, and the adsorption–degradation rate reached 86% after four cycles. Through analyses of the adsorption mechanism and the photocatalytic mechanism of the composite material, it was revealed that the enhanced adsorption was mainly attributable to the abundant surface active sites available for adsorption, pore filling, and strong interactions with oxygen function–al groups. Furthermore, compared to pure BiVO_4_ and pure MgAl–LDHs, the composite material BiVO_4_/MgAl–LDHs exhibited a smaller band gap, indicating a broader visible light absorption peak. The strong adsorption capacity of the BiVO_4_/MgAl–LDHs compo–site material enabled efficient photocatalytic degradation of MB in solution. The oxygen and metal vacancy defects generated during the layer-by-layer reconstruction process might have contributed to this, as defects can reduce the band gap and separate photo–generated charge carriers. Additionally, the MgAl–LDH particles might serve as a barrier layer for electrons and prevent recombination, thereby enhancing the spatial charge sep–aration. Finally, under the combined effects of various reactive species, the MB in solu–tion was degraded into H_2_O, CO_2_, and some intermediate products.

## Figures and Tables

**Table 1:** BET surface areas (ABET), pore volumes (VBJH), and pore diameters (DBJH) for BiVO_4_ and BiVO_4_/MgAl-LDHs.

Sample	ABET (m^2^/g)	VBJH (cm^3^/g)	DBJH (nm)
BiVO_4_	2.57 m^2^/g	0.01 cm^3^/g	14.99 nm
BiVO_4_/MgAl–LDHs	76.52 m^2^/g	0.35 cm^3^/g	16.27 nm

## Data Availability

Data will be made available on request.
